# *CsTRM5* regulates fruit shape via mediating cell division direction and cell expansion in cucumber

**DOI:** 10.1093/hr/uhad007

**Published:** 2023-01-30

**Authors:** Yang Xie, Xiaofeng Liu, Chengzhen Sun, Xiaofei Song, Xiaoli Li, Haonan Cui, Jingyu Guo, Liu Liu, Ao Ying, Zeqin Zhang, Xueyun Zhu, Liying Yan, Xiaolan Zhang

**Affiliations:** Hebei Key Laboratory of Horticultural Germplasm Excavation and Innovative Utilization, College of Horticulture Science and Technology, Hebei Normal University of Science and Technology, Qinhuangdao 066004, China; State Key Laboratories of Agrobiotechnology, Joint International Research Laboratory of Crop Molecular Breeding, Beijing Key Laboratory of Growth and Developmental Regulation for Protected Vegetable Crops, Department of Vegetable Sciences, China Agricultural University, Beijing 100193, China; Engineering Laboratory of Genetic Improvement of Horticultural Crops of Shandong Province, College of Horticulture, Qingdao Agricultural University, Qingdao 266109, China; Hebei Key Laboratory of Horticultural Germplasm Excavation and Innovative Utilization, College of Horticulture Science and Technology, Hebei Normal University of Science and Technology, Qinhuangdao 066004, China; Hebei Key Laboratory of Horticultural Germplasm Excavation and Innovative Utilization, College of Horticulture Science and Technology, Hebei Normal University of Science and Technology, Qinhuangdao 066004, China; Hebei Key Laboratory of Horticultural Germplasm Excavation and Innovative Utilization, College of Horticulture Science and Technology, Hebei Normal University of Science and Technology, Qinhuangdao 066004, China; Hebei Key Laboratory of Horticultural Germplasm Excavation and Innovative Utilization, College of Horticulture Science and Technology, Hebei Normal University of Science and Technology, Qinhuangdao 066004, China; State Key Laboratories of Agrobiotechnology, Joint International Research Laboratory of Crop Molecular Breeding, Beijing Key Laboratory of Growth and Developmental Regulation for Protected Vegetable Crops, Department of Vegetable Sciences, China Agricultural University, Beijing 100193, China; State Key Laboratories of Agrobiotechnology, Joint International Research Laboratory of Crop Molecular Breeding, Beijing Key Laboratory of Growth and Developmental Regulation for Protected Vegetable Crops, Department of Vegetable Sciences, China Agricultural University, Beijing 100193, China; State Key Laboratories of Agrobiotechnology, Joint International Research Laboratory of Crop Molecular Breeding, Beijing Key Laboratory of Growth and Developmental Regulation for Protected Vegetable Crops, Department of Vegetable Sciences, China Agricultural University, Beijing 100193, China; State Key Laboratories of Agrobiotechnology, Joint International Research Laboratory of Crop Molecular Breeding, Beijing Key Laboratory of Growth and Developmental Regulation for Protected Vegetable Crops, Department of Vegetable Sciences, China Agricultural University, Beijing 100193, China; Hebei Key Laboratory of Horticultural Germplasm Excavation and Innovative Utilization, College of Horticulture Science and Technology, Hebei Normal University of Science and Technology, Qinhuangdao 066004, China; Hebei Key Laboratory of Horticultural Germplasm Excavation and Innovative Utilization, College of Horticulture Science and Technology, Hebei Normal University of Science and Technology, Qinhuangdao 066004, China; State Key Laboratories of Agrobiotechnology, Joint International Research Laboratory of Crop Molecular Breeding, Beijing Key Laboratory of Growth and Developmental Regulation for Protected Vegetable Crops, Department of Vegetable Sciences, China Agricultural University, Beijing 100193, China

## Abstract

Fruit shape and size are important appearance and yield traits in cucumber, but the underlying genes and their regulatory mechanisms remain poorly understood. Here we identified a mutant with spherical fruits from an Ethyl Methane Sulfonate (EMS)-mutagenized library, named the *qiu* mutant. Compared with the cylindrical fruit shape in 32X (wild type), the fruit shape in *qiu* was round due to reduced fruit length and increased fruit diameter. MutMap analysis narrowed the candidate gene in the 6.47 MB range on Chr2, harboring the *FS2.1* locus reported previously. A single-nucleotide polymorphism (SNP) (11359603) causing a truncated protein of *CsaV3_2G013800*, the homolog of tomato fruit shape gene *SlTRM5*, may underlie the fruit shape variation in the *qiu* mutant. Knockout of *CsTRM5* by the CRISPR-Cas9 system confirmed that *CsaV3_2G013800/CsTRM5* was the causal gene responsible for *qiu*. Sectioning analysis showed that the spherical fruit in *qiu* resulted mainly from increased and reduced cell division along the transverse and longitudinal directions, respectively. Meanwhile, the repressed cell expansion contributed to the decreased fruit length in *qiu*. Transcriptome profiling showed that the expression levels of cell-wall-related genes and abscisic acid (ABA) pathway genes were significantly upregulated in *qiu*. Hormone measurements indicated that ABA content was greatly increased in the *qiu* mutant*.* Exogenous ABA application reduced fruit elongation by inhibiting cell expansion in cucumber. Taken together, these data suggest that *CsTRM5* regulates fruit shape by affecting cell division direction and cell expansion, and that ABA participates in the CsTRM5-mediated cell expansion during fruit elongation in cucumber.

## Introduction

Cucumber (*Cucumis sativus* L*.*) is a vegetable crop with important economic and biological value. The cucumber fruit is a kind of pepo fruit with three fused carpels and parietal placentation [1]. Unlike most fruits harvested at the mature stage, cucumber is usually harvested at an immature stage for fresh consumption or production of processed pickles 1–2 weeks after floral anthesis [2]. In cucumber, fruit length varies from 5 to 60 cm, and fruit shape varies from round to cylindrical [3, 4]. Fruit size and shape have been under intensive selection during domestication due to their large effects on fruit appearance and yield in cucumber [5].

Fruits originate from the carpel primordium and subsequently undergo ovary development and fertilization, which is required for fruit development in most species [3, 6, 7]. Cell division occurs mainly in early fruit development, whereas cell expansion continues until final fruit size and shape are reached [8–10]. Subsequently, fruit morphological development ceases and the fruit enters the ripening stage [11]. Among fruit morphogenesis processes, the rate, duration and direction of cell division, as well as isotropic and anisotropic cell expansion, contribute greatly to the final fruit shape and size [11]. In horticultural crops, using tomato as the model plant for studying fruit morphogenesis, some regulators have been identified for fruit shape and size. Among these, the WUS (WUSCHEL)–CLV3 (CLAVATA3) pathway plays essential roles in floral meristem development and locule number determination in tomato [12–15]. Tomato varieties with eight or more locules nearly always carry both the *fascinated* (*fas*) and the *locule number* (*lc*) mutation. *SlCLV3* and *SlWUS* underlie *fas* and *lc*, respectively [14, 15]. OVATE regulates gynoecium shape by controlling the cell division pattern in both the proximal–distal and the medial–lateral direction [16]. The OVATE family proteins (OFPs) interact with TONNEAU1 recruiting motif proteins (TRMs) to regulate fruit shape via antagonistic effects on fruit elongation in tomato [17]. *SUN*, which encodes a calmodulin binding protein, functions in fruit shape specification by increasing cell division in the longitudinal direction and decreasing cell division in the transverse direction of fruit [18]. In addition to cell division, dramatic enlargement in cell size led to increased fruit size [4, 10]. *Cell Size Regulator* (*CSR*), encoding an unknown protein, affects fruit size/weight by positively regulating cell enlargement in tomato [19].

In cucumber, fruit morphogenesis goes through three sequential phases: the fruit set phase, which involves ovary development before anthesis and fertilization; the cell division phase; and the cell expansion phase, which exhibits a typical sigmoidal pattern [3, 4]. Fruit shape is often described by fruit length (FL), fruit diameter (FD) and fruit shape index (FSI, the ratio of FL to FD) in cucumber [3]. Ovary shape and final fruit shape are generally highly correlated, indicating that cucumber fruit shape is determined before anthesis [20–22]. FL and FD largely describe fruit growth along proximal–distal and medial–lateral axes, respectively. A total of 42 quantitative trait loci (QTLs) were found associated with fruit shape (FS), FSI and fruit weight (FW), while only two QTLs were fine-mapped [3]. Specifically, *FS1.2* and *FS2.1* underlie the round fruit shape in WI7239 cucumber [22]. *CsSUN*, a homolog of the tomato fruit shape gene *SUN*, underlies *FS1.2* [22]. *CsTRM5*, an ortholog of *SlTRM5*, was speculated to be the candidate gene for *FS2.1* [17], but the specific functions of *CsSUN* and *CsTRM5* remained elusive in cucumber. Additionally, three genes were identified as controlling fruit length in cucumber. *SF1* (*Short Fruit 1*), encoding a RING-type E3 ligase, regulates fruit elongation by orchestrating the ethylene synthesis effect on cell division in developing fruit [23]. *SF2* (*Short Fruit 2*) encodes a histone deacetylase complex1 (HDC1) homolog that acts on fruit length by regulating cell proliferation [24]. A gain-of-function allele of MADS-box transcription factor *CsFUL1*, *CsFUL1^A^*, has a negative effect on fruit length by inhibiting cell division and expansion in Asian long cucumber [25]. So far, the genes and their regulatory mechanisms underlying fruit shape variation remain poorly understood in cucumber.

In this study, a novel cucumber mutant with spherical fruits was identified and designated as *qiu*. Mapping data showed that *CsTRM5* may underlie the fruit shape variation in *qiu*. Further analysis with the CRISPR-Cas9 knockout system confirmed that *CsTRM5* was the causal gene responsible for the spherical fruit phenotype in *qiu*. Cytological, transcriptomic, and hormone treatment analyses showed that *CsTRM5* regulated fruit shape by mediating cell division direction and cell expansion in cucumber.

## Results

### The cucumber *qiu* mutant produced shorter and wider fruits

**Figure 1:**
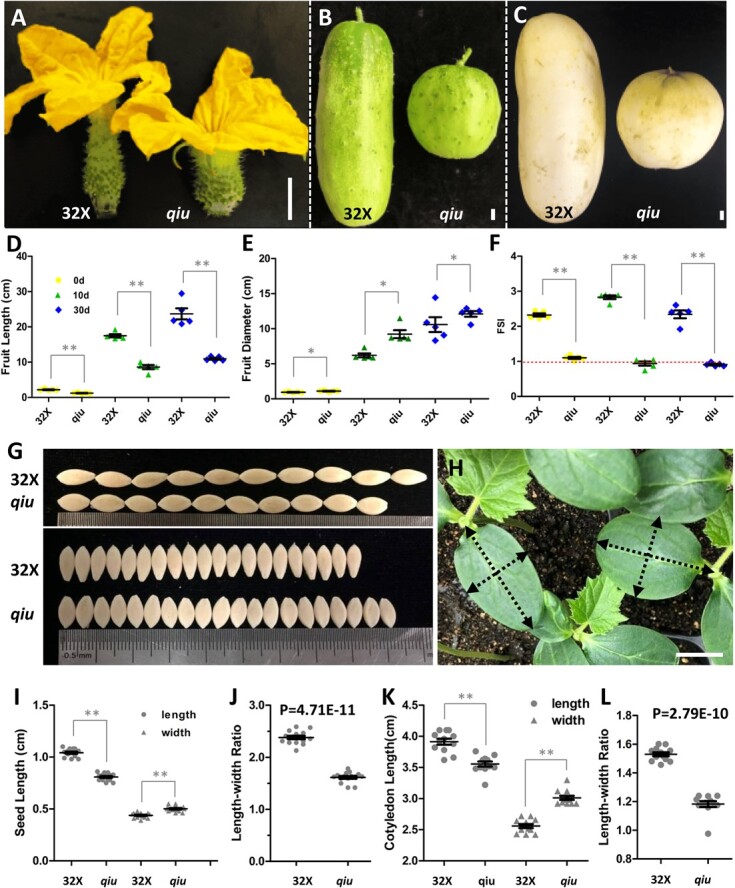
Phenotypic characterization of 32X and *qiu* mutant. **A**–**C** Fruits of 32X and *qiu* mutant at 0, 10 and 30 DAA. **D**–**F** Statistical data analysis of fruit length, diameter and FSI in 32X and *qiu* mutant. **G**, **H** Seed and cotyledon phenotypes in 32X and *qiu*. **I**, **J** Quantification data on seed length, width, and length/width ratio in 32X and *qiu*. **K**, **L** Quantification data on cotyledon length, width and length/width ratio. Scale bars: 1 cm in A–C and H.

To identify genes regulating fruit shape in cucumber, one mutant bearing ball-like fruits, named the *qiu* mutant, was chosen for further characterization from M3 lines among an EMS-mutagenized library (Supplementary Data Fig. S1A and B). Fruit development dynamics, including FL, FD, and FSI at 0, 10, and 30 DAA, were observed in wild-type (32X) and *qiu* mutant (Fig. 1A–F). Compared with 32X, the *qiu* mutant showed decreased fruit length and increased fruit diameter during different developmental stages (Fig. 1A–F). The FSI of *qiu* mutant fruit was close to 1 at 0, 10, and 30 DAA, while that of 32X was between 2.28 ± 0.29 and 2.83 ± 0.12 (Fig. 1F), and the resulting fruit shape was nearly spherical in *qiu* and cylindrical in 32X. Therefore, fruit morphogenesis is determined as early as anthesis in cucumber.

**Table 1:** Inheritance analysis of fruit shape in *qiu* mutant in cucumber.

**Population**	**Cylinder-shaped**	**Spherical-shaped**	**Segregation ratio**	***χ*** ^**2**^	***χ*** ^**2**^_**0.05**_
*F*_1_(32X × *qiu*)	15	0			
*F*_1_(*qiu*×32X)	14	0			
*F*_2_(32X × *qiu*)	125	38	3:1	0.247	3.841

The fruit shape index of cylindrical fruit (like 32X) is between 2.0 and 3.0, and that of spherical fruit (like *qiu*) is between 0.9 and 1.2.

**Figure 2:**
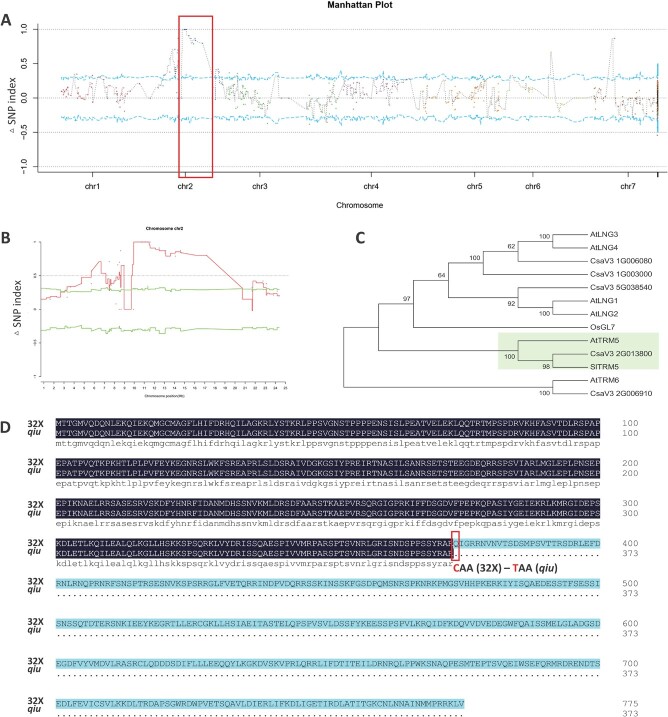
Identification of the candidate gene in *qiu* mutant. **A** MutMap analysis identified the *qiu* locus (red box) in the cucumber genome. **B** MutMap analysis identified the *qiu* locus in Chr2. **C** Phylogenetic analysis of TRMs in cucumber, *Arabidopsis*, rice, and tomato. **D** Protein sequence information on CsTRM5 in 32X and *qiu* lines. The red box indicates the changed amino acid between the 32X and *qiu* lines.

In addition to fruit phenotype, the seeds in *qiu* mutant plants became shorter and wider compared with 32X (Fig. 1G). The length and width of seeds in *qiu* were 0.81 ± 0.04 and 0.50 ± 0.03 cm, a decrease of 22% and an increase of 28% compared with 32X, respectively (Fig. 1I and J). Similarly, cotyledons displayed the same changes as fruits and seeds in *qiu* compared with 32X (Fig. 1H, K, and L). Hypocotyl length, plant height, and male/female flower size in *qiu* were smaller compared with 32X (Supplementary Data Fig. S1C–F).

### Inheritance analysis and mapping of the candidate gene for *qiu* mutant

To perform genetic analysis of *qiu*, an *F*_2_ population was constructed using 32X as the pistillate parent and *qiu* as the pollen parent. All *F*_1_ individuals by reciprocal crossing (32X × *qiu* and *qiu* × 32X) displayed a cylindrical fruit shape like that of 32X, indicating the dominance of cylindrical fruit shape over spherical fruit phenotype. Among 163 *F*_2_ individuals there was a total of 125 plants with cylindrical fruits and 38 plants with spherical fruits, representing a segregation ratio of 3:1 (*χ*^2^ = 0.247, *χ*^2^_0.05_ = 3.841) (Table 1). The results verified that the spherical fruit phenotype of *qiu* was controlled by a single recessive gene.

To identify the candidate region contributing to the spherical fruit phenotype in *qiu*, a modified MutMap was performed. After resequencing, a total of 30 277 Mb (99.27% coverage), 15 443 Mb (99.31% coverage), 17.617 Mb (99.23% coverage), and 22 844 Mb (99.22% coverage) clean reads for WT pool (32X), mutant pool (*qiu*), S-pool (lines with spherical fruit in *F*_2_), and C-pool (lines with cylindrical fruit in *F*_2_) plants were obtained, respectively (Supplementary Data Table S3). We detected 32 463 SNPs (homozygous between two parents) detected between the wild-type and mutant pools and used for SNP-index analysis. To validate correlation accuracy, we restricted the SNPs as follows: (i) those with SNP index value <0.3 and SNP depth <7 in both the C-pool and the S-pool were filtered out; (ii) those with a missing SNP index in the C-pool or S-pool were filtered out. After filtering, 8345 SNVs (single-nucleotide variants) were used for *Δ*SNP-index analysis and plotting. A total of 90 SNVs harboring a high *Δ*SNP index (out of 95% confidence values; SNP index >0.7 in S-pool and <0.3 in C-pool; SNP index >0.7 in C-pool and <0.3 in S-pool) were identified within the whole genome (Supplementary Data Table S4), and resulted in three peak regions (Fig. 2A). Among them, one peak harbored multiple SNVs located at 10.71–17.18 Mb on Chr2 and had high quality; the other two peaks each harbored only one SNV in Chr6 or Chr7 and had low confidence (Fig. 2A). Therefore, the peak in Chr2 was named as the *qiu* locus, which harbored the reported *FS2.1* [22]. SNP annotation results showed that there were 11 SNPs in the exon region causing amino acid change within the *qiu* locus (Supplementary Data Table S5). Among them, only two SNPs (11359603 and 14075205) were located in the coding regions of cucumber functional proteins, and the remaining nine SNPs, encoding proteins of fungi or bacteria, were not mapped to the cucumber genome and thus were not considered as the candidate sites for *qiu* locus. The SNP from G to A (11359603) caused a premature stop codon and a severely truncated protein (from 776 to 373 amino acids) of *CsaV3_2G013800*, which encoded the homolog of SlTRM5 associated with fruit shape in tomato (Fig. 2C and D). The other SNP (14075205), a C-to-T transition from 32X to the *qiu* line, causing an alanine-to-threonine change in *CsaV3_2G016800*, encoded a WD40 repeat-like superfamily protein (Supplementary Data Table S5). Further protein sequence analysis showed that this amino acid is not conserved between species (Supplementary Data Fig. S2A). Expression analysis showed that the expression level of both *CsaV3_2G013800* and *CsaV3_2G016800* was unchanged in ovaries of 32X and *qiu* (Supplementary Data Fig. S2B and C). In conclusion, we assumed that *CsaV3_2G013800* may be the candidate gene for *qiu* and the *FS2.1* locus, and named it *CsTRM5*.

### Temporal and spatial expression pattern of *CsTRM5*

**Figure 3:**
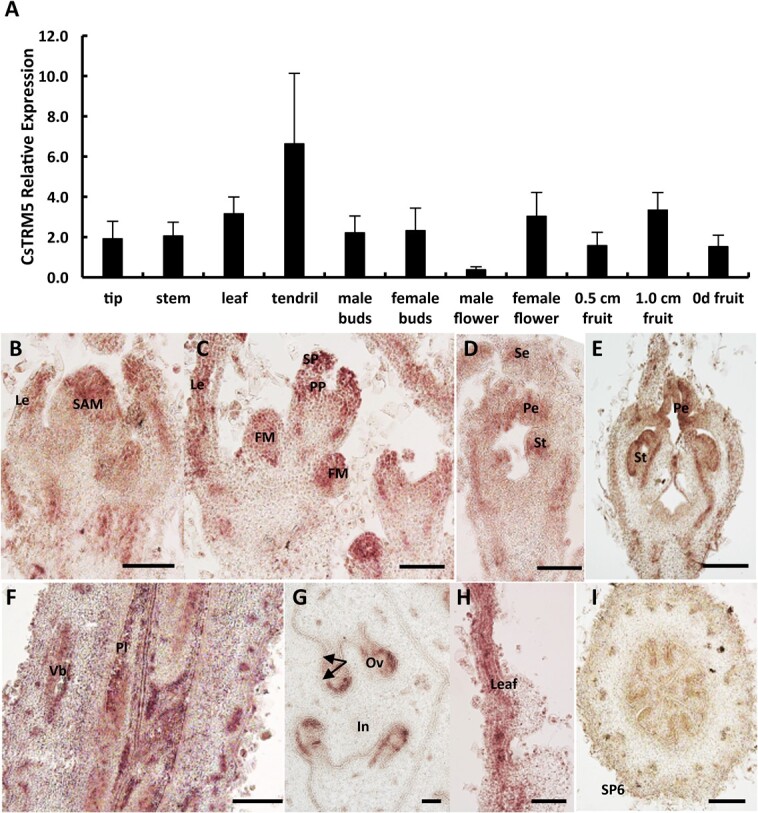
Expression pattern analysis of *CsTRM5* in cucumber*.***A** Expression levels of *CsTRM5* in different organs of 32X line detected by qRT–PCR. **B**–**H***In situ* hybridization of *CsTRM5* in cucumber shoot tip (**B**), leaf (**H**), and floral organs (**C**–**G**). **I**, Negative control of *CsTRM5* sense probe in fruit cross-section. Le, leaf; SAM, shoot apical meristem; FM, floral meristem; SP, sepal primordium; PP, petal primordium; Se, sepal; Pe, petal; St, stamen; Vb, vascular bundle; Pl, placenta; Ov, ovule; In, integument. Scale bars: 100 μm.

To explore the expression pattern of *CsTRM5*, qRT–PCR analysis was performed in different organs, including tip, stem, leaf, tendril, flower buds, flowers, and fruits at different stages in cucumber. *CsTRM5* displayed a universal expression pattern (Fig. 3A). *In situ* hybridization showed that *CsTRM5* was expressed in shoot apical meristem, floral meristem, leaf, and young flower buds (Fig. 3B–H). *CsTRM5* transcripts were detected in sepal, petal, and stamen primordia in young flower buds (Fig. 3C–E). Among male flower buds, *CsTRM5* was expressed in petal and stamen (Fig. 3E). *CsTRM5* was expressed in whole fruit and highly expressed in placenta at stage 9 of female flower buds (Fig. 3F). Subsequently *CsTRM5* signals were mainly enriched in ovule integuments (Fig. 3G). No signal was detected upon hybridization with the sense *CsTRM5* probe (Fig. 3I).

### Knockout of *CsTRM5* resulted in decreased fruit shape index in cucumber

**Figure 4:**
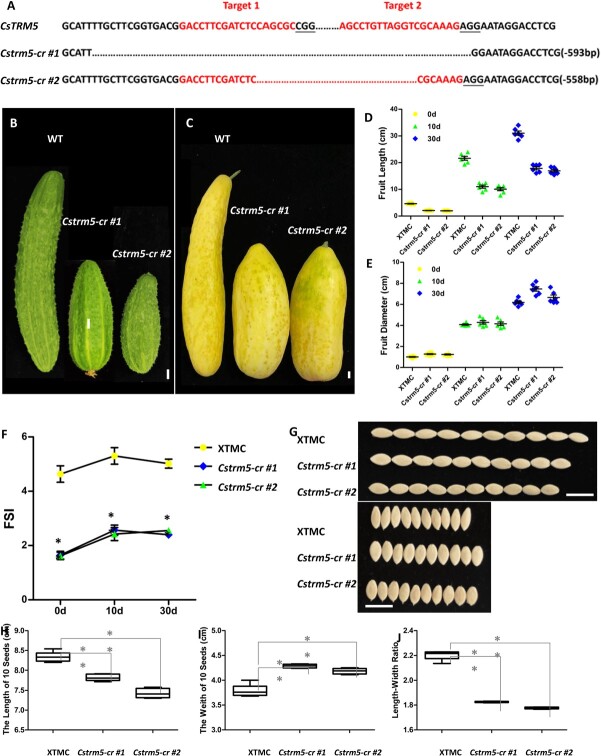
Mutation forms and phenotypes of *Cstrm5-cr* lines. **A** Mutation forms of two homozygous *T*_1_ transgenic *Cstrm5-cr* lines obtained using the CRISPR-Cas9 system. **B**, **C** Fruits at 10 and 30 DAA in XTMC and *Cstrm5-cr* lines. **D**, **E** Fruit length and diameter of XTMC and *Cstrm5-cr* lines. **F** FSI in XTMC and *Cstrm5-cr* lines. **G** Seed phenotype in XTMC and *Cstrm5-cr* lines. **H**–**J** Seed length, width, and length/width ratio in XTMC and *Cstrm5-cr* lines. Scale bars: 1 cm in **B，C** and G.

In order to confirm the function of *CsTRM5* in cucumber, we constructed *CsTRM5* knockout lines in XTMC using the CRISPR-Cas9 gene editing system. The two targets were located on the second and third exons, respectively (Supplementary Data Fig. S3A). Two homozygous loss-of-function mutant lines (*Cstrm5-cr* #1 and #2) were obtained for further characterization (Fig. 4A). Both *Cstrm5-cr* #1 and *Cstrm5-cr* #2 produced fruits with decreased length and increased diameter, resulting in a change in FSI from 4.9–5.6 in control to 2.3–2.9 in mutant lines (Fig. 4B–F), consistent with the phenotype of *qiu* and 32X (Fig. 1A–E). Similarly, the length/width ratio of seeds was also decreased, by 17 and 19% in *Cstrm5-cr* #1 and *Cstrm5-cr* #2, respectively, compared with XTMC (Fig. 4G–J). These data suggested that *CsTRM* regulates fruit and seed shape in cucumber.

### Mutation in *CsTRM5* led to change in cell division direction in fruits

**Figure 5:**
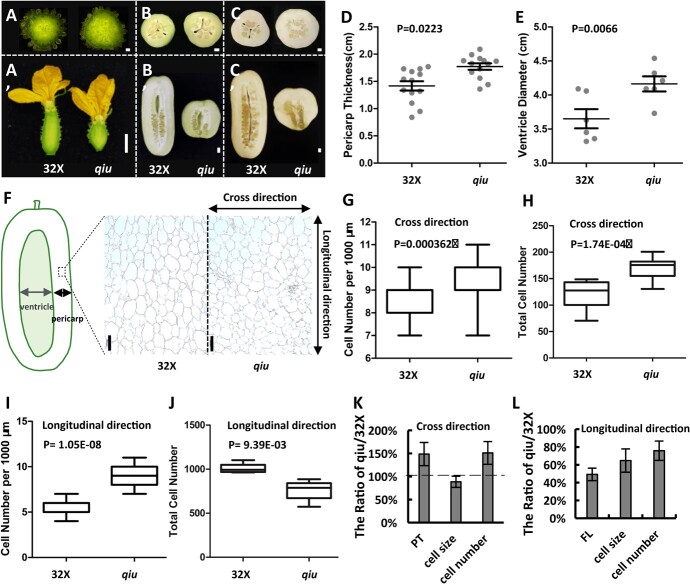
Cell division direction and cell expansion were changed in the *qiu* mutant. **A**–**C** Cross-sections and longitudinal sections of fruits at 0, 10 and 30 DAA in 32X and *qiu* mutant. **D**, **E** Pericarp thickness and ventricle diameter of fruit at 10 DAA in 32X and *qiu*. **F** Longitudinal paraffin section of fruit at 10 DAA in 32X and *qiu*. Gray double-headed arrow indicates ventricle diameter; black double-headed arrow indicates pericarp thickness. **G**, **H** Pericarp cell number per 1000 μm and total cell number in cross direction. **I**, **J**, Pericarp cell number per 1000 μm and total cell number in longitudinal direction. **K**, **L** PT, FL, cell size, and cell number ratio of *qiu* to 32X in cross (K) or longitudinal direction (L). Scale bars: 1 cm in **A**–**C**, 200 μm in **F**.

In both *qiu* and *Cstrm5-cr* lines, fruit length and diameter changed (Figs 1A–E and 4B–E). Further characterization of fruit internal morphogenesis during fruit development indicated that *qiu* fruit exhibited larger pericarp thickness (1.77 ± 0.21 cm in *qiu*, 1.42 ± 0.31 cm in 32X) and ventricle diameter (4.16 ± 0.27 cm in *qiu*, 3.65 ± 0.34 cm in 32X) than wild-type fruit (Fig. 5A–E), indicating that both pericarp and ventricle had a positive effect on the increase in fruit diameter. Locule number generally influences fruit diameter and size [1]. We checked the locule number of 32X and *qiu* and found that both possessed three locules (Fig. 5A–C), suggesting that locule number was not the cause of the increased diameter in *qiu* mutant.

To further explore the cellular basis of the change in FSI, we evaluated pericarp thickness, cell size and cell number of the pericarp in the longitudinal and cross directions. Cucumber fruit can be roughly estimated as a cylinder, so the pericarp has the same length as the fruit. We dissected fruits longitudinally at 10 DAA and investigated the cell number per unit length in the cross and longitudinal directions in the pericarp. The cell number per unit length was increased in both the cross (9.6 ± 1.0 in *qiu*, 8.4 ± 0.7 in 32X) and the longitudinal (8.6 ± 1.1 in *qiu*, 5.8 ± 0.8 in 32X) direction, indicating that the cell size became smaller in *qiu* compared with 32X (Fig. 5G and I). We counted higher cell numbers in the cross direction in *qiu* (170.1 ± 20.1) than in 32X (119.2 ± 25.4) (Fig. 5D and F–H), indicating that there was more cell proliferation in the cross direction in the pericarp of *qiu*. Combining fruit length with cell number per unit length along the longitudinal direction, the total cell numbers were decreased in the longitudinal direction in *qiu* (762.3 ± 108.5) than in 32X (1005.2 ± 53.0) (Figs 3D and 5F, I, and J), suggesting less cell proliferation in the longitudinal direction. In addition, we calculated the proportions of pericarp thickness (PT), FL, cell size and cell number in *qiu* relative to 32X in both cross and longitudinal directions (Fig. 5K and L). The data showed that the increased PT in *qiu* is directly caused by the increase in cell number in the cross direction, and the decreased FL in *qiu* is caused by both reduced cell size and decreased cell number in the longitudinal direction (Fig. 5K and L). To sum up, cell division in the pericarp was enhanced in the cross direction and repressed in the longitudinal direction in *qiu* compared with 32X, while cell expansion was inhibited in both the cross and the longitudinal direction in *qiu*.

Likewise, we evaluated pericarp thickness and cell size and number in the longitudinal and cross directions in XTMC and *Cstrm5-cr* lines (Supplementary Data Fig. S3B–I). The relative data between XTMC and *Cstrm5-cr* lines displayed similar trends to that in 32X and *qiu* (Supplementary Data Fig. S3B–I and Fig. 5). Cell size became smaller in *Cstrm5-cr* lines in both the cross and the longitudinal direction, whereas cell number increased in the cross direction and decreased in the longitudinal direction in *Cstrm5-cr* lines compared with the wild-type (Supplementary Data Fig. S3B–I). These data suggested that *CsTRM5* regulated fruit shape by functioning in the cell division direction and cell expansion in cucumber, in which the change in cell division direction plays a vital role.

### The abscisic acid pathway was involved in fruit shape regulation by *CsTRM5* in cucumber

**Figure 6:**
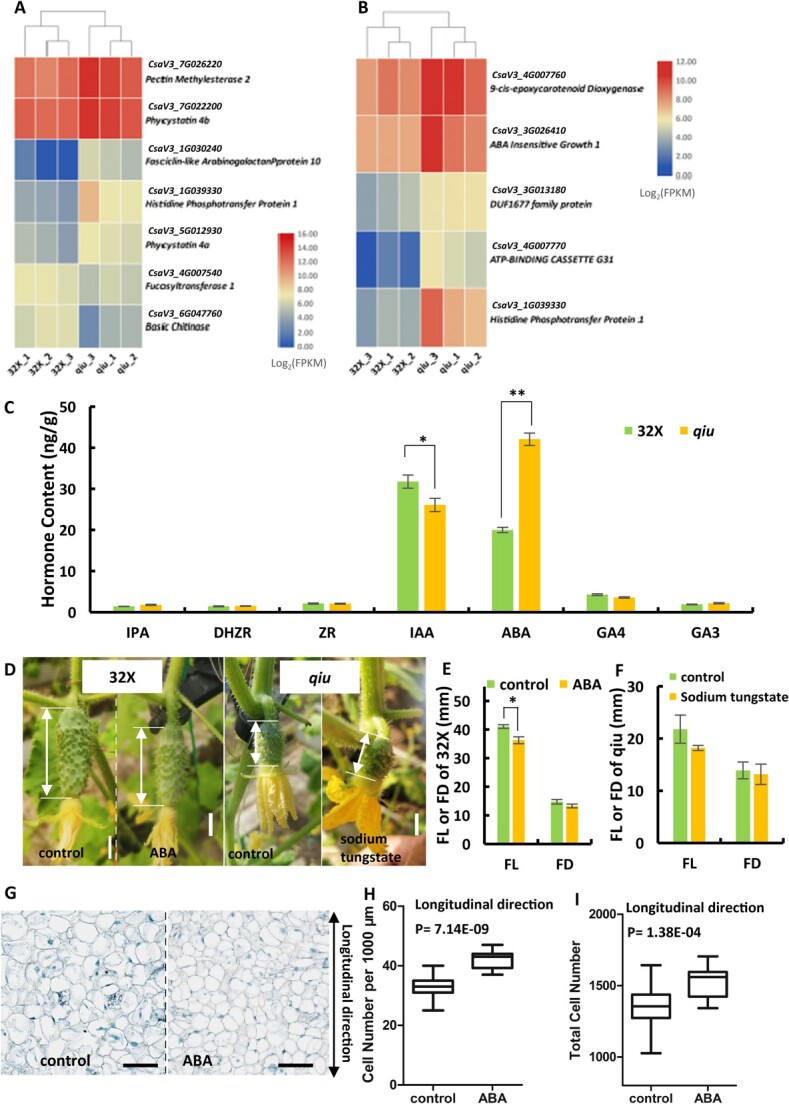
ABA plays an important role in fruit length variation in cucumber. **A**, **B** DEGs relative to cell wall development (**A**) and hormone (**B**) pathways between *qiu* and 32X lines. **C** Hormone content of young fruits in *qiu* and 32X lines. **D** Fruit phenotype of 32X and *qiu* lines after ABA or sodium tungstate treatment. **E**, **F** Change in FL and FD after ABA (**E**) or sodium tungstate (**F**) treatment. **G** Longitudinal section of fruit at 3 DAA in 32X after ABA treatment. **H**, **I** Pericarp cell number per 1000 μm and total cell number in longitudinal direction. Scale bars: 1 cm in **D**, 100 μm in **G**.

To identify putative downstream targets of *CsTRM5*, RNA-seq of young fruits/ovaries at 0 DAA between 32X and *qiu* lines was performed and 43 DEGs were obtained, of which 28 were upregulated and 15 were downregulated (Supplementary Data Table S6). Cell elongation in plants requires addition and rearrangements of cell wall components. Among these DEGs, we examined seven genes that were reported to regulate cell wall activities (Fig. 6A). For example, *CsaV3_1G030240*, encoding a fasciclin-like arabinogalactan protein, displayed a 46.4-fold (log_2_ fold change = 5.54) increase in the *qiu* mutant (Supplementary Data Table S6). In *Arabidopsis*, fasciclin-like arabinogalactans (FLAs) contributed to the cell wall matrix by affecting cellulose deposition [38]. *CsaV3_7G026220* displayed a 3.74-fold increase in the mutant and encoded a pectin methylesterase affecting cell elongation by modifying the cell wall [39]. In addition, DEG analysis showed that ABA- and cytokinin-related genes were significantly upregulated in *qiu* compared with those in 32X (Fig. 6B). ABA is the major regulator of senescence and stress and plays an important role in cell wall modifications, while cytokinin is involved in cell division [40, 41]. *CsaV3_4G007760* was increased 3.4-fold in the *qiu* mutant and encoded 9-*cis*-epoxycarotenoid dioxygenase, whose homolog in *Arabidopsis* is a key enzyme in the biosynthesis of ABA; *CsaV3_4G007770* was increased 16.27-fold in the *qiu* mutant, it encoded an ABCG-type transporter participating in ABA transport; *CsaV3_3G026410* and *CsaV3_3G013180* were increased 3.92- and 3.71-fold in the *qiu* mutant, respectively, and both function in the ABA signal pathway (Fig. 6B) [42–44]. qRT–PCR results validated the elevated expression of the above four ABA-related genes in young fruits of *qiu* compared with 32X (Supplementary Data Fig. S4). In addition, *CsaV3_1G039330*, a homolog of AHP (*Arabidopsis thaliana* histidine phosphotransfer protein), which positively regulates cytokinin signaling [45], was increased 22.59-fold in *qiu* compared with wild-type (Fig. 6B).

To further explore the relationship between CsTRM5 and hormone pathways, ovaries at the green bud stage from *qiu* and 32X were used for hormone measurements. Our data showed that the IAA level was significantly decreased, while ABA content was dramatically increased in *qiu* compared with 32X (Fig. 6C). Contents of cytokinin were not changed (Fig. 6C). IAA was shown to promote cell expansion by inducing cell-wall acidification in plants [46, 47]. In cucumber, auxin was reported to mediate fruit length and fruit neck elongation by cell division and cell expansion [25, 48]. In *qiu*, the reduced IAA content was consistent with smaller cell size and reduced FL in fruit (Fig. 5F–H). ABA is well known for mediating plant responses to abiotic stress by participating in cell expansion [49]. To verify the role of ABA in fruit shape regulation in cucumber, exogenous treatment with ABA or ABA biosynthesis inhibitor (sodium tungstate) was performed in 32X and *qiu*, respectively. Our data showed that fruit elongation in 32X was significantly reduced upon ABA treatment compared with control fruits (Fig. 6D and E). However, upon treatment with sodium tungstate, both FL and FD of *qiu* displayed no significant changes (Fig. 6D–F). Sectioning and statistical analysis showed that the cell size was significantly reduced in 32X after ABA treatment (Fig. 6G and H), suggesting that ABA was involved in the CsTRM5-mediated cell expansion in the longitudinal direction. Additionally, total cell number in 32X after ABA treatment was increased in longitudinal direction (Fig. 6I), not like the decreased total cell number in* qiu* compered to 32X (Fig. 5J), implying that ABA may not participate in CsTRM5-mediated cell division in cucumber.

## Discussion

### *CsTRM5* was responsible for *qiu* mutant with spherical fruit shape

Recent studies have revealed that TRM family members control the shape of fruit, seed and leaf in tomato, *Arabidopsis*, and tobacco [17, 50–54]. TONNEAU1 (TON1) proteins share similarity with a human centrosomal protein and TRM1-TON1 recruitment is essential for microtubule organization in the cortex [30]. Plant microtubule arrays are involved in the direction of cell division and expansion [30]. In *Arabidopsis*, *LONGIFOLIA1* (*LNG1*) and *LONGIFOLIA2* (*LNG2*) promote longitudinal polar cell elongation to regulate leaf morphology [50]. *LNG1*/*2*/*3*/*4* acted redundantly on leaf longitudinal growth by changing turgor pressure and activating XTHs (xyloglucan endotransglucosylase/hydrolases) [51]. PIF4 can induce thermomorphogenic growth by promoting *LNG1*/*2* expression levels [55]. *SLG7* (*Slender Grain on Chromosome 7*), encoding a protein homologous to AtLNG1 and AtLNG2, produces slender grains [52], and copy number variation of *GL7* (*Grain Length on Chromosome 7*) contributes to the grain size diversity in rice [54]. In tomato, TRMs interact with OVATE family proteins to regulate cell division patterns in fruit shape by co-locating microtubules [17]. In cucumber, *FS2.1* was mapped into 10 candidate genes, including a homolog of AtTRM5/SlTRM5 [17]. All these TRMs belong to the *Arabidopsis* LONGIFOLIA (TRM1–5) clade, indicating that LONGIFOLIA subclade members participated in organ morphology by changing cell division and cell elongation in different plant species.

In this study, we identified a novel mutant, *qiu*, with spherical fruits from an EMS-mutagenesis library. Inheritance analysis of *qiu* showed that the spherical fruit phenotype was controlled by a single recessive gene (Table 1). A modified MutMap method was used for mapping the *qiu* locus, which was delimited to a 6.47-MB region harboring the *FS2.1* locus. In this region, one SNP caused a truncated protein of *CsaV3_2G013800* (Supplementary Data Table S5). The phylogenetic analysis showed that CsaV3_2G013800 is an ortholog of AtTRM5, sharing 34.23% identity. In a previous study, *CsTRM5* (*CsaV3_2G013800*/*Csa2G227860*) was also presumed to be the candidate gene for spherical fruit shape in the *FS2.1* locus [17]. In this study, we speculated that *CsaV3_2G013800* was the candidate gene for the spherical fruit shape in *qiu* and *FS2.1*. Considering the low transgenic efficiency of 32X, we constructed knockout lines of *CsaV3_2G013800* using the CRISPR-Cas9 system in the XTMC inbred line. We found that the fruits of *Cstrm5-cr* lines were shorter and thicker, consistent with the change in the fruits of *qiu*. But fruit shape in *Cstrm5-cr* lines was a short cylinder, not round like *qiu*. This may be due to the fact that XTMC has a larger FSI than 32X. The FSI of *Cstrm5-cr* lines was half of that of XTMC, consistent with the FSI of *qiu* being about half of that of 32X (Figs 1F and 4F). Except for fruit shape, the change in seed and cotyledon shape in *Cstrm5-cr* lines is consistent with those in *qiu*. The above phenotypic analysis confirmed that *CsaV3_2G013800*/*CsTRM5* was responsible for the spherical fruit shape in *qiu*.

### *CsTRM5* affects fruit shape by regulating cell division direction and cell expansion in cucumber

The fruit is a complex organ that develops along three axes: proximal–distal, medial–lateral, and abaxial–adaxial [56, 57]. Growth in the proximal–distal and medial–lateral axes is reflected in fruit length (longitudinal growth) and fruit diameter (cross growth), respectively. In cucumber, Pan *et al*. [3] summarized the fruit development dynamics of 11 genotypes from 6 days before anthesis to 30 DAA, including four lines with long cylindrical fruits, three lines with short cylindrical fruits, and four lines with spherical fruits. All these lines showed the typical sigmoidal pattern of FL and FD, and FSI of 0 DAA fruit and final mature fruit (30 DAA fruit) exhibited a high correlation (*r* = 0.9876), indicating that pre-anthesis factors play important roles in specifying fruit shape in cucumber [3]. The rate, duration, and direction of cell division, as well as isotropic and anisotropic cell expansion, together determined fruit shape and size [11, 17, 58].

The dynamics of fruit development at three points, including 0 DAA (ovary stage), 10 DAA (commercial fruit stage), and 30 DAA (mature fruit stage), were investigated in this study to examine fruit shape change between *qiu* and 32X. The results showed that fruit shape was determined at the pre-anthesis stage. The smaller cell size in the cross direction and larger fruit diameter in *qiu* indicated that its fruit growth along the medial–lateral axis is mainly due to enhanced cell proliferation in this direction (Fig. 5). Combining data on cell size along the longitudinal direction and fruit length of *qiu* and 32X, it can be concluded that both cell division and cell expansion were reduced along the proximal–distal direction in *qiu*. Similar data were found in *Cstrm5-cr* knockout lines (Supplementary Data Fig. S3). These findings suggested that *CsTRM5* regulates fruit shape by modifying the cell division direction and cell expansion in cucumber. In tomato, *Sltrm5* could rescue the fruit elongation phenotype of the double mutant *ovate*/*suppressor of ovate* (*sov1*) by antagonizing the role in cell division pattern of the proximal portion of the fruit in the proximo–distal direction, while the *trm5* mutant alone produced fruits with similar shape to that of wild-type [17]. In cucumber, the fruit length variations were much greater than in tomato. Consistently, the effect of *CsTRM5* on fruit shape regulation was much more dramatic than that of *SlTRM5*, suggesting the crucial role of *CsTRM5* in fruit shape determination in cucumber.

### Abscisic acid may be involved in CsTRM5-mediated fruit elongation in cucumber

Plant hormones are closely related to fruit shape. During the fruit morphogenesis process, cell division and cell expansion contribute greatly to final fruit shape and size [11]. In this study, among 43 DEGs between 32X and *qiu*, four ABA-related genes, and one cytokinin-related gene were identified (Fig. 6B). One key enzyme (*CsaV3_4G007760*) in the biosynthesis of ABA and one ABA transporter (*CsaV3_4G007770*) in *qiu* were upregulated ~3.4- and ~16.3-fold over 32X (Supplementary Data Table S6). The ABA content was dramatically increased in *qiu* compared with 32X (Fig. 6C), implying that ABA may act downstream of CsTRM5 during fruit shape determination. ABA is well known as the major player in senescence and stress through cell wall modifications [59]. Cell elongation in plants requires addition and rearrangements of cell wall components [60]. Cellulose and pectin are components of the cell wall. Seven genes regulating cell wall activities were identified among DEGs, including *FLA* (*CsaV3_1G030240*) affecting cellulose deposition, and *CsaV3_7G026220*, modifying the cell wall as a pectin methylesterase [38, 39]. *CsTRM5* is a member of the LONGIFOLIA subclade, whose member *LNG3*/*4* could activate cell wall modification-related genes to affect cell elongation [51].

Exogenous ABA treatment resulted in reduced fruit elongation in 32X by inhibiting cell size in the longitudinal direction, but displayed no effect on fruit diameter (Fig. 6D–H). Unlike the decreased total cell number in *qiu* in the longitudinal direction compared to 32X (Fig. 5J), the total cell number in 32X was increased after ABA treatment (Fig. 6I). These results indicated that ABA is involved in CsTRM5-mediated cell expansion during fruit elongation, but not in the regulation of cell division direction during fruit shape formation. Whether and how IAA or other hormone pathways play roles in the CsTRM5-mediated cell division direction need further studies in cucumber.

## Materials and methods

### Plant materials and growth conditions

The material of 32X (South China type cucumber) with short cylindrical fruits was a high-generation inbred line. The mutant *qiu* with spherical fruits was discovered among M3 lines in an Ethyl Methane Sulfonate (EMS)-mutagenized population of the 32X line. The 32X line as female parent was crossed with *qiu* as male parent to develop 15 *F*_1_-1 plants and the reciprocal cross produced 14 *F*_1_-2 plants. A total of 163 *F*_2_ plants were produced by self-crossing of *F*_1_-1 for an inheritance study, genotyping, and gene identification. Also used in the study was XTMC (North China type), with long fruits, also a high-generation inbred line. The materials used in this study were planted according to standard practices in greenhouses in China Agricultural University, Beijing, or in Hebei Normal University of Science and Technology, Qinhuangdao.

### Phenotype analysis

Fruit measurements were taken of three fruits between the 15th and 25th nodes of each plant, and five or more plants were measured. To ensure sufficient seed set, open flowers were tagged and hand-pollinated. Seed length and width were measured using 20 seeds. For cotyledon shape analysis, cotyledons were collected from 10 or more seedlings at the one true leaf stage.

### Identification of the candidate gene for *qiu* by MutMap

A modified MutMap method was used to identify the candidate gene for *qiu.* A total of 20 lines with cylindrical fruits and 20 lines with spherical fruits were selected from the *F*_2_ population. DNA was extracted from each plant and mixed in equal amounts to construct the cylindrical fruit bulk (C-pool) and the spherical fruit mutant bulk (S-pool). DNA from the two parental plants (20 individuals) was extracted to construct the wild-type pool (*P*_1_, 32X) and the mutant pool (*P*_2_, *qiu*), respectively. DNA from the two parental plants, C-pool and S-pool, were re-sequenced with Illumina HiSeq TMPE150. The raw reads were filtered using the NGSQC toolkit software [26]. BWA was used to align the clean reads to the reference genome [27], and single-nucleotide polymorphisms (SNPs) were detected using the Genome Analysis Toolkit (GATK) [28]. ANNOVAR software was used for functional annotation for SNP detection results [29].

### Phylogenetic tree construction

*Arabidopsis* TRM proteins (TRM1, TRM2, TRM3, TRM4, TRM5, TRM6) and the homologs in tomato, rice, and cucumber were used for phylogenetic tree analysis [30]. Protein sequences of TRMs from cucumber were obtained using BLAST tools in CuGenDB (Chinese Long_V3.0). All protein sequences were aligned and constructed using MEGA6.0 by the neighbor-joining method. Accession numbers of TRM sequences used for phylogenetic analysis are provided in Supplementary Data Table S1.

### Quantitative real-time PCR

Plants at blooming and fruit-bearing stage were used for sampling under the long-day condition. The young shoot tip, stem, leaf, tendril, male buds, female buds, male flower, female flower, fruit at anthesis, and fruits of 0.5 and 1.0 cm were sampled for RNA extraction using Trizol reagent, then reverse-transcribed to cDNA using the TianScript II RT Kit (Tiangen, China). Quantitative real-time PCR (qRT–PCR) was performed using TB Green™ Premix Ex Taq™ (Takara, Japan) on the Bio-Rad CFX384 system. *UBIQUITIN EXTENSION PROTEIN* (*UBI-EP*, *Csa000874*) was used as the internal reference gene [31]. Three biological replicates and three technical replicates were performed for each gene. The primer information is listed in Supplementary Data Table S2.

### Paraffin sectioning

Fruit mesocarps were sampled along the longitudinal direction with 3.7% formalin–acetic acid–alcohol (FAA) overnight, dehydrated with a grade series of ethanol, infiltrated with xylene, and then embedded in paraffin (Leica, 39 601 095, Germany). The sections (10 μm) were used for cell morphology observation by deparaffinating and rehydration under a D72 light microscope (Olympus).

### *In situ* hybridization

The 20-day-old shoot tips and male and female flower buds were fixed with 3.7% FAA, then embedded in paraffin. Sense and antisense probes were amplified with gene-specific primers (Supplementary Data Table S2) using SP6 and T7 RNA polymerase with the DIG RNA Labeling Kit (SP6/T7) (Roche, USA), respectively. Sample sectioning and hybridization were performed as described previously [32].

### Cucumber transformation

Two target sequences (19 bp) located in the second and third exons were used for construction of a CRISPR-Cas9 knockout vector (pKSE402 with GFP fluorescent screening marker pCBC-DT1T2 as an intermediate vector) (Supplementary Data Table S2). The recombinant knockout vector was transformed into *Agrobacterium tumefaciens* EHA105. The XTMC line, with long fruits, had the same *CsTRM5* genotype as the 32X line and a well-established CRISPR system, and was thus used as transgenic background material [33]. Cotyledons at 36 hours after germination as the explants were infected with EHA105 (in infection liquid with OD_600_ = 0.2–0.3) under negative pressure. After 3 days of co-culture in darkness, explants were transferred to bud differentiation medium with Timentin (200 μg/l) under 16 hours light/8 hours dark at 26°C for 3–4 weeks. Then the buds with GFP were selected and excised from explant to rooting medium. Homozygous *T*_1_ mutants without vector (GFP-free) were identified from *T*_0_ transgenic lines for further phenotype observation and data statistics. The detailed cucumber transformation protocol was as described previously [34]. Primer information is listed in Supplementary Data Table S2.

### Transcriptome analysis

Fruits at 0 days after anthesis (DAA) from 32X and *qiu* were collected for RNA-seq analysis. Three biological replicates were performed. RNA-seq libraries were constructed using the NEB Next Ultra 488 Directional RNA Library Prep Kit (NEB, USA), then loaded on to an Illumina HiSeq 4000 platform to generate 150-bp paired-end reads. The cut-offs for differentially expressed genes (DEGs) were a change in expression of at least 2.0-fold and a false discovery rate (FDR) of <0.05. The Gene Ontology (GO) terms or categories with a *P*-value <.05 were identified as significantly enriched. Sequencing data were deposited in the NCBI Gene Expression Omnibus database (https://www.ncbi.nlm.nih.gov/geo/) under accession number PRJNA804348.

### Hormone quantification by enzyme-linked immunosorbent assay

Ovaries at green bud stage of *qiu* and 32X were harvested to determine endogenous hormone contents, including indoleacetic acid (IAA), abscisic acid (ABA), gibberellic acids (GA3 and GA4), and three types of cytokinin: indolepropionic acid (IPA), *trans*-zeatin-riboside (ZR), and dihydrozeatinauxin (DHZR). About 0.5 g of fresh samples was collected and homogenized in 2 ml of 80% methanol. Extraction and hormone quantification were performed using the enzyme-linked immunosorbent assay (ELISA) following the protocol described in previous reports [35, 36]. Three biological replicates were performed for each genotype.

### Exogenous abscisic acid and abscisic acid inhibitor application

ABA and its synthesis inhibitor sodium tungstate were used as described previously [37]. 32X was treated with 50 ABA or 0 (control) μmol/l ABA (Shanghai Yuanye Bio-Technology Co., Ltd, CAS#14375-45-2), a synthetic ABA. Meanwhile, *qiu* was treated with 3 or 0 (control) mmol/l sodium tungstate (Tianjin Oubokai Chemical Co. LTD, CAS#10213-10-2). The ovary and corolla were sprayed once at anthesis and fruit measurement was performed 3 days after treatment. The results are the means ± SE of at least three replicates.

## Acknowledgements

This work was supported by the National Natural Science Foundation of China (31772327 and 32025033), the Key R&D Program of Hebei Province (21326309D), the Scientific Research Foundation of Hebei Normal University of Science and Technology (2019YB015 and 2020JK004), and the Natural Science Foundation of Hebei Province (C2020407015).

## Author contributions

X.Zhang, L.Y., and X.Liu designed the research; Y.X., X.Liu, and C.S. performed the experiments; X.Zhang, X.Liu, and Y.X. wrote the paper; X.S., X.Li, H.C., J.G., L.L., A.Y., Z.Z., and X.Zhu provided experimental assistance; all authors revised the manuscript.

## Data availability

The datasets have been submitted to the NCBI-SRA database with the BioProject ID PRJNA804348.

## Conflict of interest statement

The authors declare no conflict of interests.

## Supplementary data

Supplementary data is available at *Horticulture Research* online.

## Supplementary Material

Web_Material_uhad007
