# Supplementary material for: CsTRM5 regulates fruit shape via mediating cell division direction and cell expansion in cucumber
Source: Hortic Res. 2023 Jan 30;10(3):uhad007. doi: 10.1093/hr/uhad007 (PMC10028494; doi:10.1093/hr/uhad007)
Supplement: Web_Material_uhad007 [file web_material_uhad007.zip › TRM-supplymental figures-R2y-f.pptx]

## Slide 1
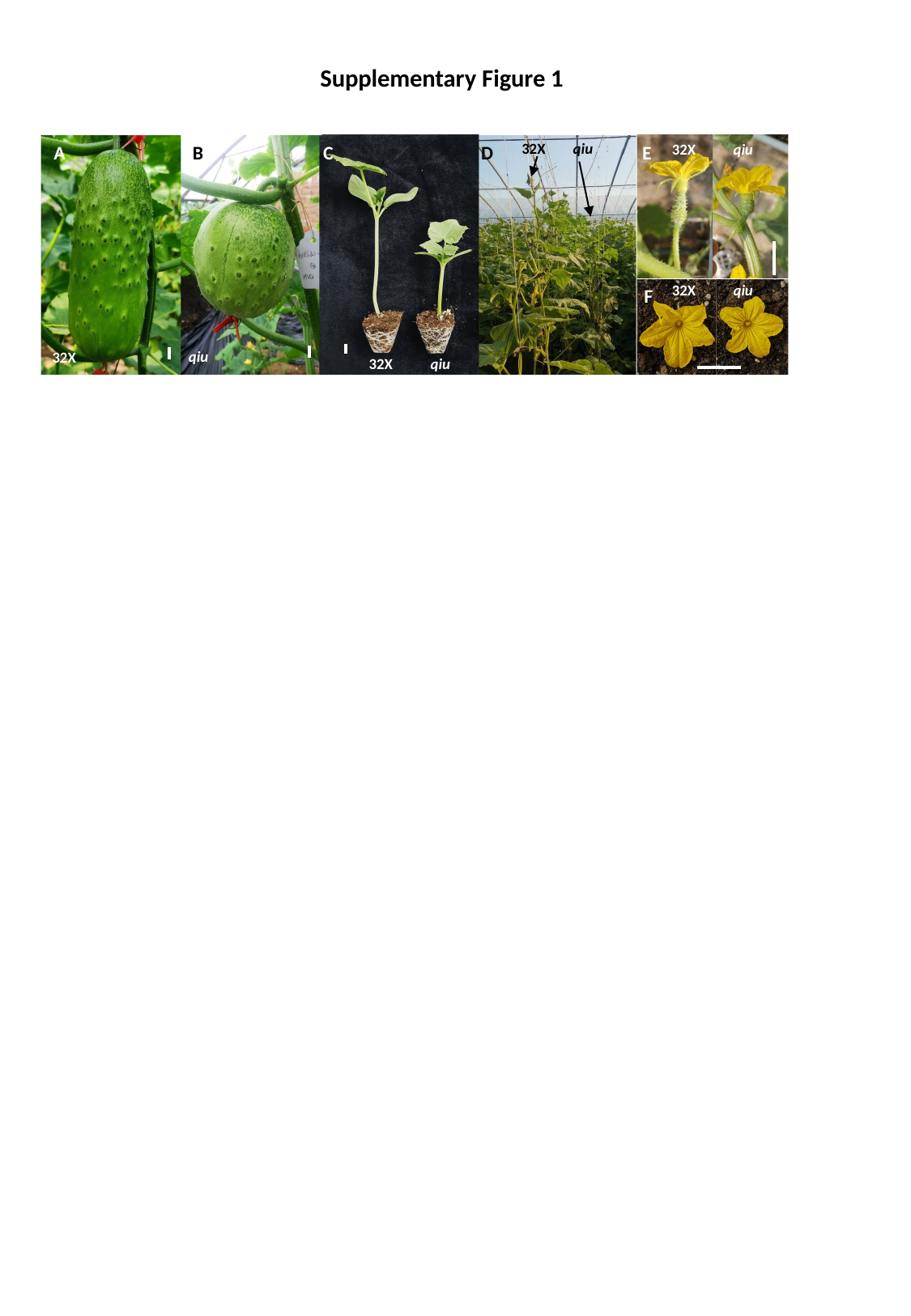

Supplementary Figure 1
32X qiu
32X qiu
E
A
B
qiu
32X
C
D
32X qiu
F
32X qiu

## Slide 2
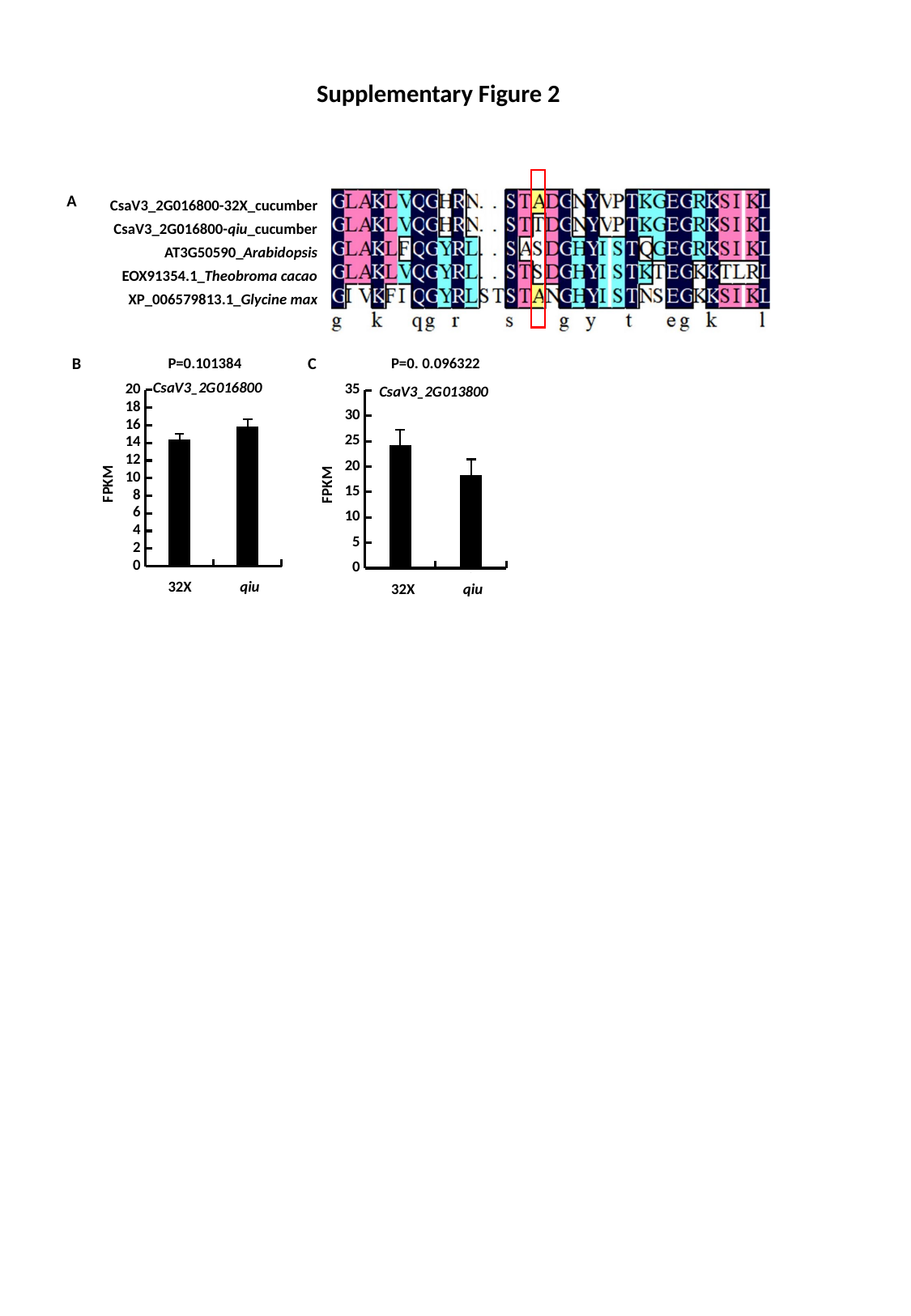

Supplementary Figure 2
A
CsaV3_2G016800-32X_cucumber
CsaV3_2G016800-qiu_cucumber
AT3G50590_Arabidopsis
EOX91354.1_Theobroma cacao
XP_006579813.1_Glycine max
### Chart:
| Category | CsaV3_2G013800 |
|---|---|
| 32X | 24.27241203 |
| qiu | 18.3552922 |
### Chart:
| Category | CsaV3_2G016800 |
|---|---|
| 32X | 14.41618675 |
| qiu | 15.84240481 |P=0.101384
P=0. 0.096322
B
C
32X qiu
32X qiu

## Slide 3
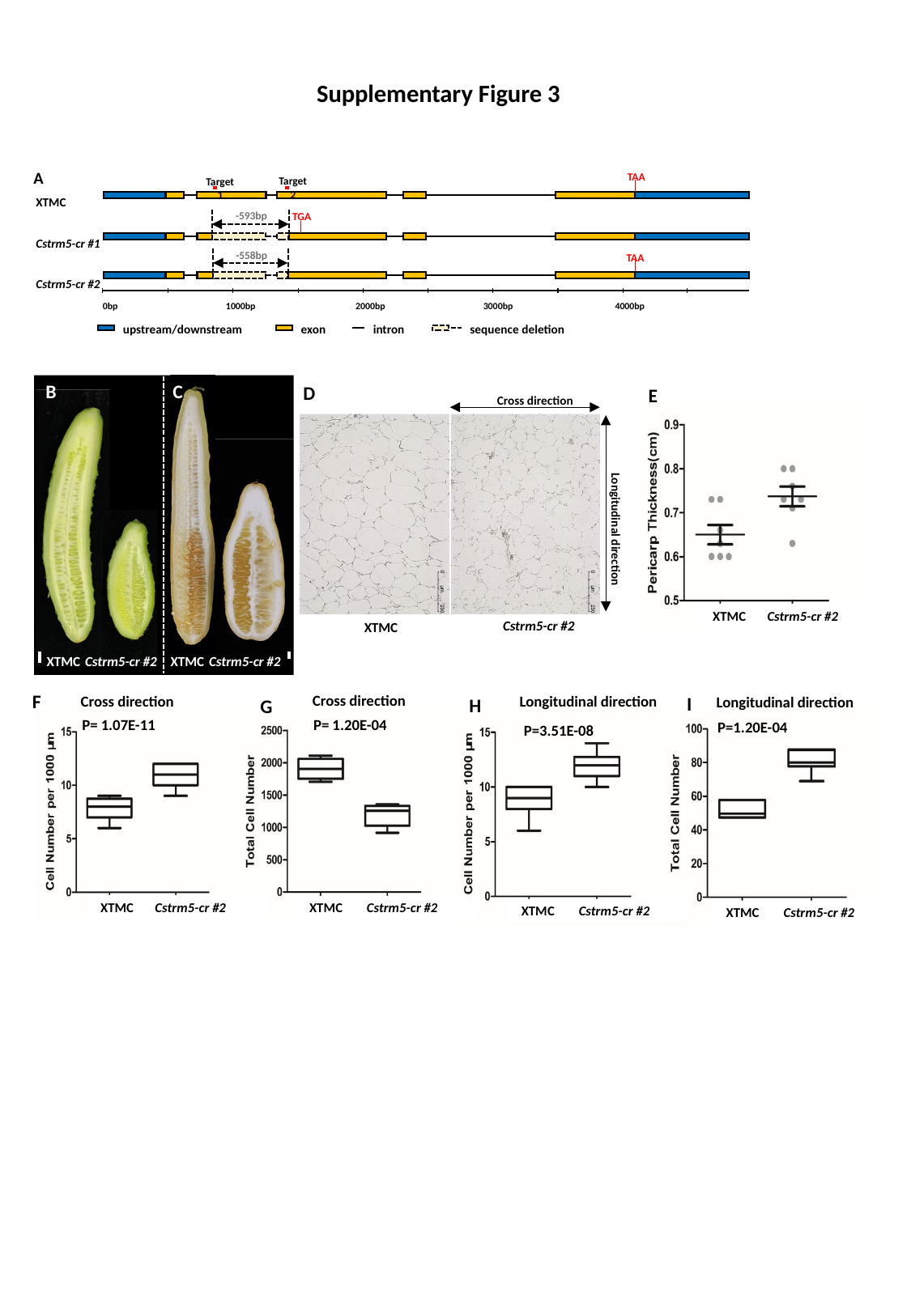

Supplementary Figure 3
XTMC
Cstrm5-cr #1
Cstrm5-cr #2
TAA
-593bp
TGA
-558bp
TAA
0bp
1000bp
2000bp
3000bp
4000bp
upstream/downstream
exon
intron
sequence deletion
A
Target 2
Target 1
B
C
XTMC
Cstrm5-cr #2
XTMC
Cstrm5-cr #2
D
Cross direction
Longitudinal direction
Cstrm5-cr #2
XTMC
E
XTMC Cstrm5-cr #2
F
Cross direction
Longitudinal direction
Cross direction
I
Longitudinal direction
H
G
P= 1.07E-11
P= 1.20E-04
P=1.20E-04
P=3.51E-08
XTMC Cstrm5-cr #2
XTMC Cstrm5-cr #2
XTMC Cstrm5-cr #2
XTMC Cstrm5-cr #2

## Slide 4
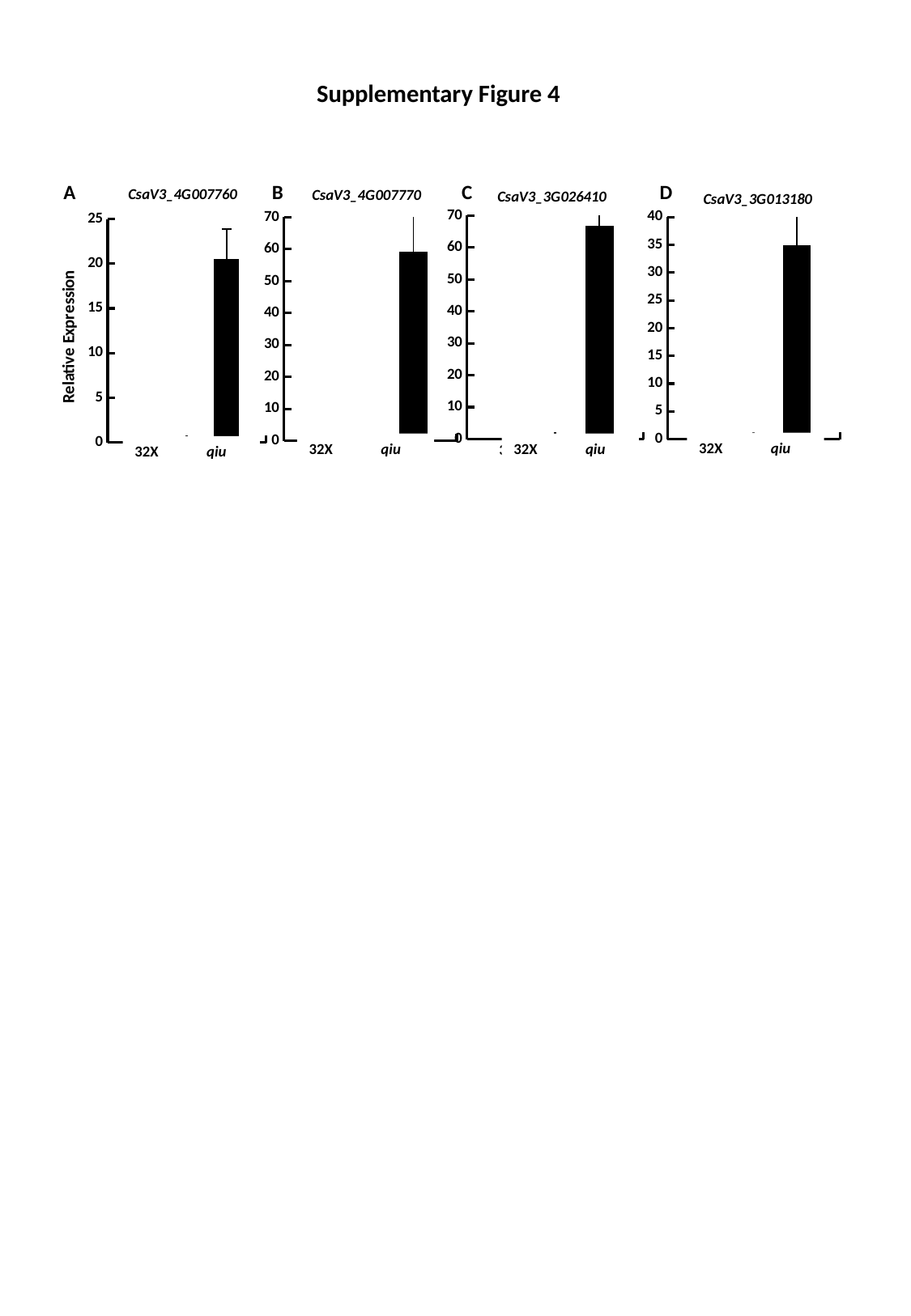

Supplementary Figure 4
### Chart:
| Category | CsaV3_3G026410 |
|---|---|
| 32X | 0.20524594260527232 |
| qiu | 66.57912049760296 |
### Chart:
| Category | CsaV3_4G007770 |
|---|---|
| 32X | 0.09997476218157375 |
| qiu | 59.14277802497372 |
### Chart:
| Category | CsaV3_3G013180 |
|---|---|
| 32X | 0.3918604465618151 |
| qiu | 34.794911374127224 |
### Chart:
| Category | CsaV3_4G007760 |
|---|---|
| 32X | 0.2821569881429708 |
| qiu | 20.492268987060758 |A
B
C
D
32X qiu
32X qiu
32X qiu
32X qiu
